# The Mitochondria-Independent Cytotoxic Effect of Leflunomide on RPMI-8226 Multiple Myeloma Cell Line

**DOI:** 10.3390/molecules26185653

**Published:** 2021-09-17

**Authors:** Grzegorz Adamczuk, Ewelina Humeniuk, Magdalena Iwan, Dorota Natorska-Chomicka, Kamila Adamczuk, Agnieszka Korga-Plewko

**Affiliations:** 1 Independent Medical Biology Unit, Faculty of Pharmacy, Medical University of Lublin, 20-093 Lublin, Poland; ewelinahumeniuk@umlub.pl (E.H.); agnieszkakorga@umlub.pl (A.K.-P.); 2 Department of Toxicology, Faculty of Pharmacy, Medical University of Lublin, 20-093 Lublin, Poland; magda.iwan@umlub.pl (M.I.); dorota.chomicka@umlub.pl (D.N.-C.); 3 Department of Biochemistry and Molecular Biology, Faculty of Medicine, Medical University of Lublin, 20-093 Lublin, Poland; kamilaadamczuk@umlub.pl

**Keywords:** leflunomide, teriflunomide, multiple myeloma, dehydrogenase dihydroorotate, protein tyrosine kinases, mitochondria

## Abstract

Leflunomide, an anti-inflammatory agent, has been shown to be effective in multiple myeloma (MM) treatment; however, the mechanism of this phenomenon has not been fully elucidated. The aim of the study was to assess the role of mitochondria and dihydroorotate dehydrogenase (DHODH) inhibition in the cytotoxicity of leflunomide in relation to the MM cell line RPMI 8226. The cytotoxic effect of teriflunomide—an active metabolite of leflunomide—was determined using MTT assay, apoptosis detection, and cell cycle analysis. To evaluate DHODH-dependent toxicity, the cultures treated with teriflunomide were supplemented with uridine. Additionally, the level of cellular thiols as oxidative stress symptom was measured as well as mitochondrial membrane potential and protein tyrosine kinases (PTK) activity. The localization of the compound in cell compartments was examined using HPLC method. Teriflunomide cytotoxicity was not abolished in uridine presence. Observed apoptosis occurred in a mitochondria-independent manner, there was also no decrease in cellular thiols level. Teriflunomide arrested cell cycle in the G2/M phase which is not typical for DHODH deficiency. PTK activity was decreased only at the highest drug concentration. Interestingly, teriflunomide was not detected in the mitochondria. The aforementioned results indicate DHODH- and mitochondria-independent mechanism of leflunomide toxicity against RPMI 8226 cell line.

## 1. Introduction

**Figure 1:**
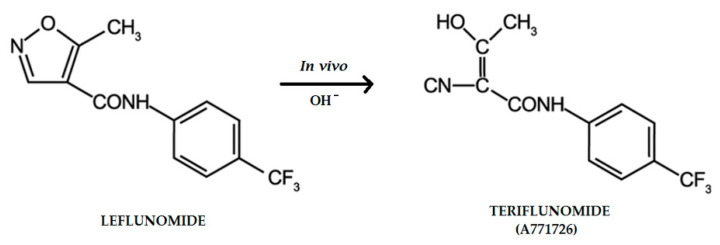
Chemical structures of leflunomide and its active metabolite—teriflunomide.

Leflunomide is anti-inflammatory and immunomodulatory drug which was approved for treatment of autoimmune diseases. Especially, it is used to reduce the symptoms of rheumatoid arthritis and psoriatic arthritis [1,2]. It is well absorbed after oral administration and it is almost completely converted during the first pass. Leflunomide is metabolized to one major metabolite A771726 (teriflunomide), which is responsible for its activity in vivo, and many other minor metabolites (Figure 1) [3].

**Figure 2:**
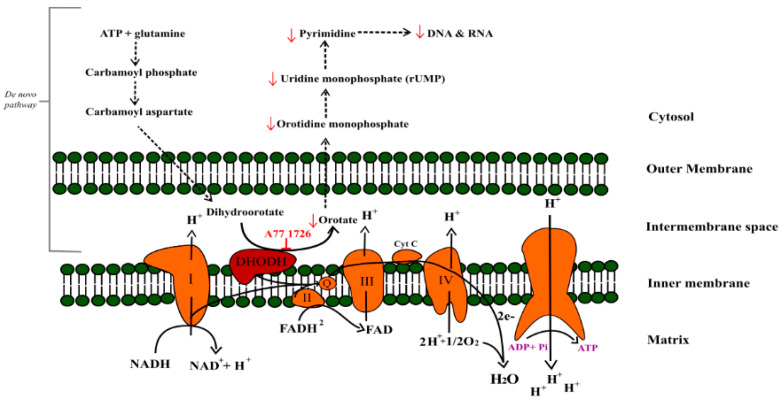
Graphical representation of the inhibition effect of teriflunomide on the de novo synthesis of pyrimidine nucleotides via dihydroorotate dehydrogenase (DHODH). DHODH links de novo synthesis of pyrimidine nucleotides to oxidative phosphorylation by means of CoQ, its electron acceptor.

The primary mechanism of action is inhibition of de novo pyrimidine synthesis by targeting dihydroorotate dehydrogenase (DHODH). DHODH is the fourth of six enzymes in the biosynthesis pathway of pyrimidines. As opposed to other enzymes of this pathway, DHODH is located on the outer surface of the inner mitochondrial membrane (Figure 2). DHODH converts L-dihydroorotate (DHO) to orotate (ORO). Via this conversion, DHODH is associated with the electron transport chain. The resulting electron from oxidation of DHO is transferred to ubiquinone, and subsequently to cytochrome c oxidase [4,5,6]. In this way, inhibition of DHODH contributes to the reduction of the level of pyrimidine nucleotides necessary for the synthesis of RNA and DNA in a tumor cell in addition to disrupting the function of mitochondria [7,8,9,10]. Another known mechanism of action of leflunomide is the inhibition of tyrosine kinase activity associated with cytokines and growth factor receptors [4,11,12]. The aforementioned mechanisms are associated with the intensity of cell proliferation and thus they may indicate the possible anticancer activity of leflunomide.

In recent years, leflunomide has been shown to be cytotoxic in relation to several cancer cell lines: bladder cancer [13], head and neck cancers [14], melanoma [15], leukemia [16,17], and thyroid cancer [18]. Currently, the most advanced research on leflunomide includes multiple myeloma (MM). In 2015, the National Cancer Institute (NCI) and City of Hope Medical Center in California started clinical trials for patients with relapsed or refractory multiple myeloma (NCT02509052). These phase I/II trial studies are currently ongoing, and participants are receiving a treatment, but new potential participants are not currently being recruited. The results of the first published phase I study showed that the use of leflunomide resulted in low toxicity with simultaneous stable disease in over 90% of patients [19]. Multiple myeloma is classified as a malignant tumor of hematological origin that arises from plasma cell overproduction [20]. In 2020, there were over 170,000 incidences of multiple myeloma worldwide (GLOBOCAN 2020). Treatment of MM is primarily based on reducing the population of clonal plasma cells, thus decreasing the symptoms of the disease. Despite great progress in medicine and constantly emerging new therapeutic methods, in most cases multiple myeloma is a recurrent and incurable disease.

Although leflunomide has been shown to be effective in studies, the exact mechanism of its cytotoxic activity in MM has not been fully elucidated and the role of DHODH inhibition remains uncertain. Here, the aim of the study was to assess the role of DHODH in the cytotoxicity of leflunomide in relation to the multiple myeloma cell line RPMI 8226.

## 2. Results

### 2.1. Cytotoxicity of Teriflunomide

**Figure 3:**
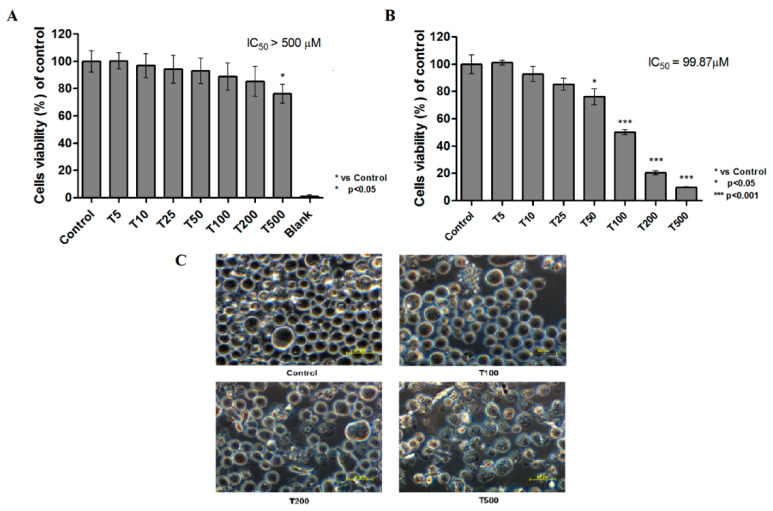
Relative viability of (**A**) skin fibroblast cell line—BJ; and (**B**) RPMI 8226 cell line treated with teriflunomide (T) (5–500 μM) or DMSO as vehicle in control cultures for 24 h determined by MTT assay. ‘Blank’ presents results for mixture of medium without cells, MTT and DMSO. The results were calculated as % of control cultures viability which were averaged to define the 100%. Values were presented as mean ± SD derived from three independent experiments. (**C**) RPMI 8226 cells morphology analyzed under a phase-contrast microscope Nikon Eclipse Ti. The cells were treated with teriflunomide (T) (100–500 µM) or DMSO as vehicle in control cultures for 24 h (magnification ×600, scale bar = 50 µm).

The human skin fibroblasts BJ were used to evaluate cytotoxicity of teriflunomide in tested concentration against normal cells. Cytotoxicity was evaluated by the MTT assay after 24 h of incubation. Teriflunomide was not highly cytotoxic to the BJ cell line at tested concentrations (Figure 3A). A slight decrease of cell viability was noted only at the highest compound concentration. The IC_50_ value for teriflunomide was not achieved.

Teriflunomide was significantly toxic to RPMI 8226 cell line in concentration of 50 µM and higher, IC50 = 99.87 µM (Figure 3B). For further studies 100, 200, and 500 µM concentrations of compound were chosen.

Consistent with the MTT assay, examination of morphological changes revealed cell death following tested compound treatment (Figure 3C). Cells treated with teriflunomide were shriveled as compared to control cells, and contained cytoplasmic blebbing. Teriflunomide contributed to chromatin condensation in MM cells. The observed changes in cell morphology increased with increasing teriflunomide concentrations.

**Figure 4:**
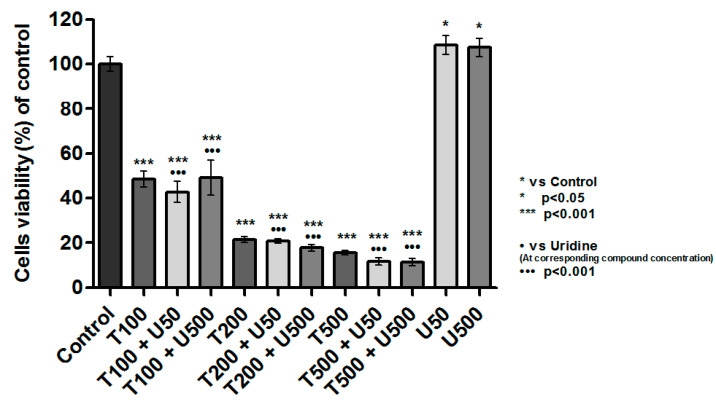
Relative multiple myeloma cell line RPMI 8226 viability treated with teriflunomide (T) (100–500 µM) and uridine (U) (50 and 500 µM) for 24 h determined by MTT assay. The results were calculated as % of control cultures viability which were averaged to define the 100%. Values were presented as mean ± SD derived from three independent experiments.

To test whether the observed toxicity of teriflunomide is related to DHODH inhibition, cells were treated simultaneously with the drug and uridine. The addition of uridine did not result in the reversal of the effect of teriflunomide on the cells, regardless of concentration (Figure 4). There were no significant differences between viability of cells treated with teriflunomide and teriflunomide (in the corresponding concentration) with uridine.

### 2.2. Teriflunomide-Induced Cell Cycle G2/M Phase Arrest

**Figure 5:**
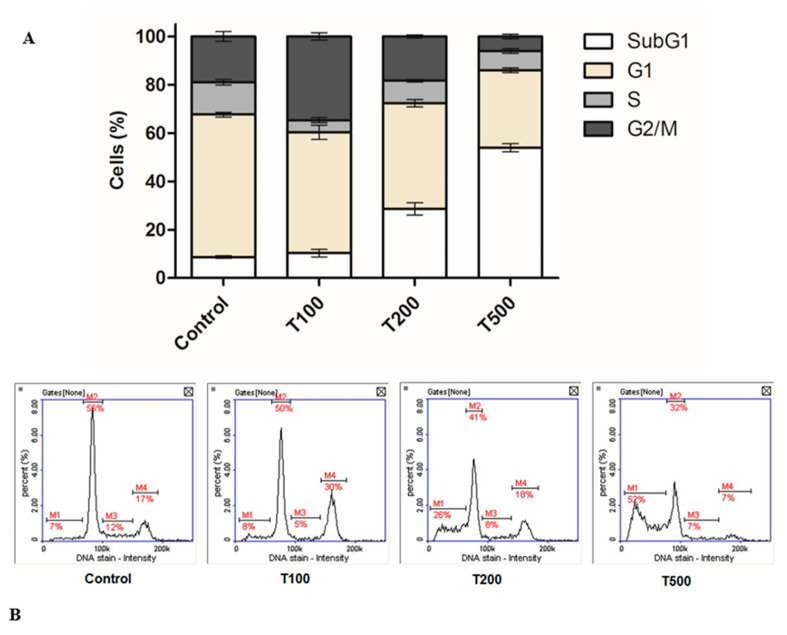
(**A**) Cell cycle analysis by image cytometry. The results were calculated as % of control values which were averaged to define the 100%. The RPMI 8226 cells were treated with Teriflunomide (T) (100–500 µM) or DMSO as vehicle in control cultures for 24 h. Values were presented as mean ± SD derived from three independent experiments. (**B**) Representative histograms (M1-4: cell cycle phases subG1, G1, S, G2/M, respectively).

The analysis of cell cycle revealed that incubation of cells with teriflunomide at concentration of 100 µM resulted in G2/M phase arrest (Figure 5). In case of higher concentrations of teriflunomide (200 µM and 500 µM) the percentage of cells in the sub G1 phase was elevated to over 25% and 50% respectively.

### 2.3. Teriflunomide-Induced RPMI 8226 Cells Apoptosis

**Figure 6:**
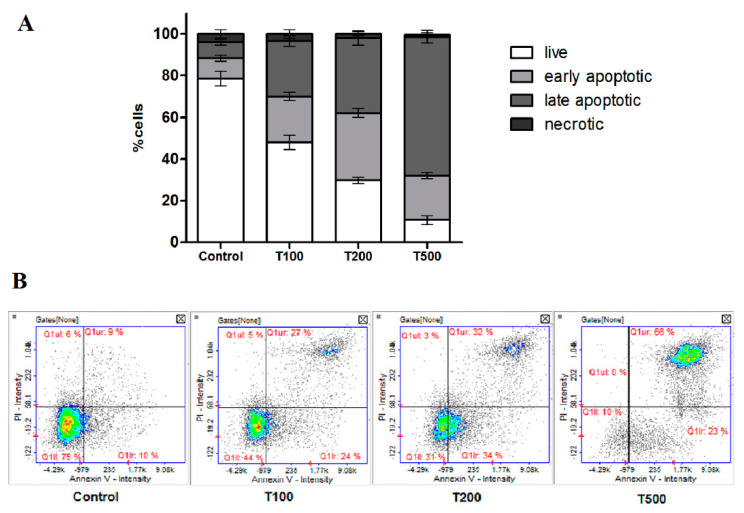
(**A**) Detection of cell apoptosis/necrosis in RPMI 8226 cells with annexin V-FITC and propidium iodide staining using image cytometry. The MM cells were treated with teriflunomide (T) (100–500 µM) or DMSO as vehicle in control cultures for 24 h. Values were presented as mean ± SD derived from three independent experiments. (**B**) The results show one representative experiment of three independently performed. Q1II-live, Q1lr- early apoptotic, Q1ur- late apoptotic, and Q1uI- necrotic cells.

The analysis of apoptosis confirmed the cytotoxic effect of teriflunomide in tested concentrations in RPMI 8226 cells, and also revealed that cells observed in cell cycle analysis in sub G1 phase of cell cycle were clearly apoptotic. The assay showed that, with increasing concentration of teriflunomide, the number of cells in early- and late-apoptotic phase increased (Figure 6). At 500 µM of teriflunomide, over 60% of the cell population was in late-apoptosis stage and approximately 20% was in the early apoptotic stage.

### 2.4. No Effect of Teriflunomide on the Level of Cellular Thiols

**Figure 7:**
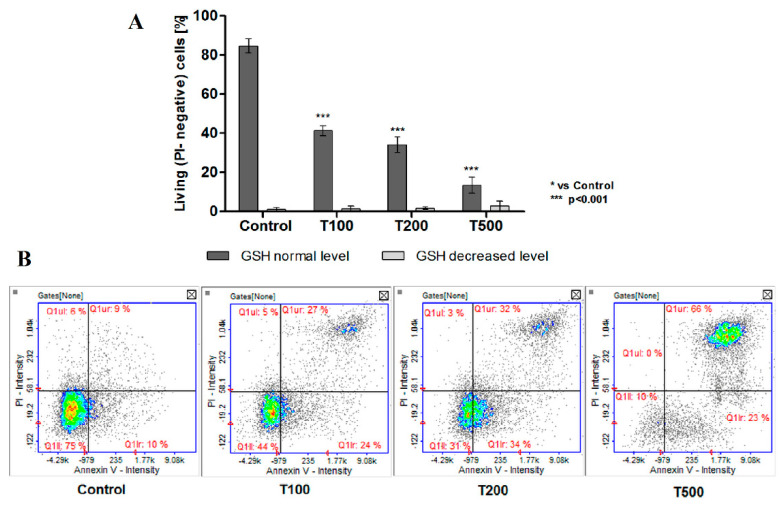
(**A**) Thiols levels in RPMI 8226 cells treated with teriflunomide (T) (100–500 µM) or DMSO as vehicle in control cultures based on image cytometry analysis. Scatter plots show the VB-48™ intensity versus the intensity of PI. Values were presented as mean ± SD derived from three independent experiments. (**B**) The results show one representative experiment of three independently performed. Q1II—PI negative cells with decreased GSH level, Q1Ir—healthy cells, Q1ur—dead cells.

To test whether teriflunomide induces oxidative stress as a results of DHODH inhibition the level of cellular thiols were measured. Thiols (mainly reduced glutathione—GSH) are key components involved in the maintenance of redox balance, which possess the ability to remove reactive oxygen species (ROS) The changes in the level of cellular thiols was not observed in case of any teriflunomide concentrations (Figure 7). The thiols level did not differ from the level observed in the control cells. On the representative scatter plots, it can be observed that teriflunomide did not reduce the VB48 intensity in cells. As expected on the basis of cell cycle and apoptosis analysis, the growing populations of PI-stained cells were observed, thus confirming the cytotoxic effect of the tested compound.

### 2.5. Teriflunomide-Induced Mitochondria—Independent Apoptosis in RPMI 8226 Cells

**Figure 8:**
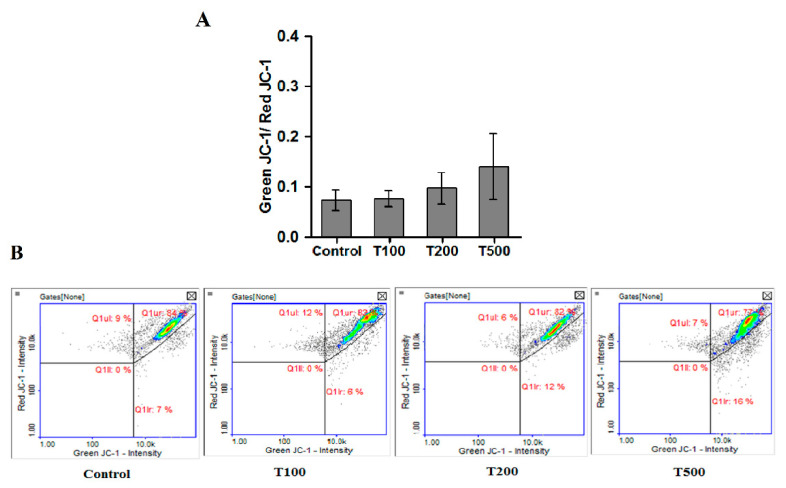
(**A**) Mitochondrial membrane potential in RPMI 8226 cells treated with teriflunomide (T) (100–500 µM) or DMSO as vehicle in control cultures based on image cytometry analysis. Scatter plots show the Green JC-1 intensity versus the intensity of Red JC-1. Values were presented as mean ± SD derived from three independent experiments. (**B**) The results show one representative experiment of three independently performed.

In order to evaluate mitochondrial involvement in apoptosis due treatment with teriflunomide in RPMI 8226 cells the mitochondrial membrane potential assay was performed. Teriflunomide did not change the mitochondrial membrane potential in RPMI 8226 cells at any tested concentrations (Figure 8). The mitochondrial membrane potential did not differ from the potential observed in the control cells. Even in 500 µM of teriflunomide, decrease of mitochondrial membrane potential was not considerable.

### 2.6. Teriflunomide Inhibited Activity of Protein Tyrosine Kinases

**Figure 9:**
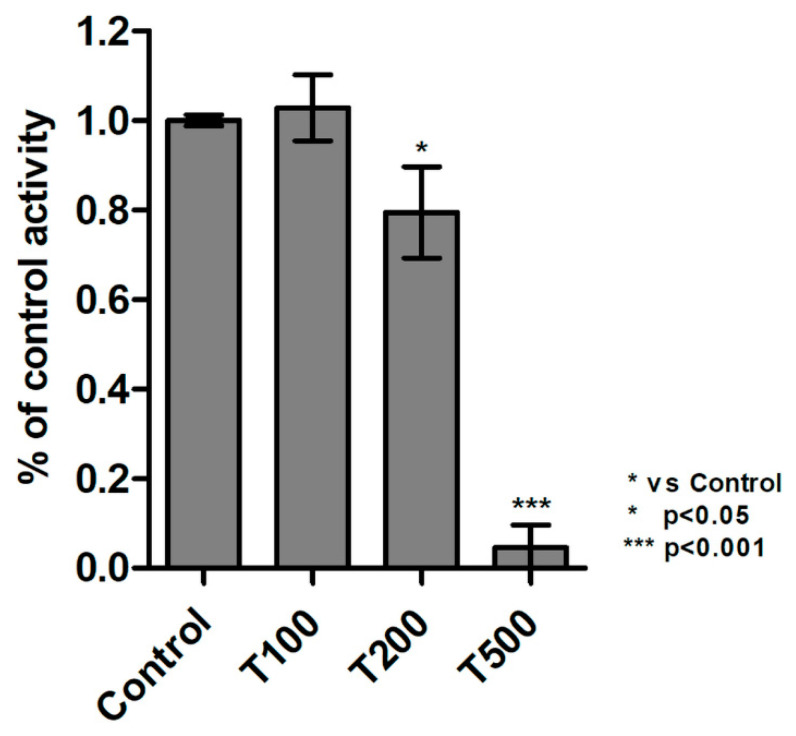
Results of protein tyrosine kinases (PTK) activity assay. The results were calculated as % of control values which were averaged to define the 100%. The values of PTK in samples were standardized to the viability of cells obtained in MTT assay. The RPMI 8226 cells were treated with teriflunomide (T) (100–500 µM) or DMSO as vehicle in control cultures for 24 h. Values were presented as mean ± SD derived from three independent experiments.

The measurement of protein tyrosine kinases activity (PTK) revealed that teriflunomide at 100 µM concentration did not change the activity of tyrosine kinases in RPMI 8226 cell as compared to control cells (Figure 9). Slight decrease in PTK activity was observed in cells treated with teriflunomide at 200 µM. At the highest concentration—500 µM—the drug contributed to an almost complete reduction of PTK activity.

### 2.7. No Effect of Teriflunomide on the DHODH Gene Expression

**Figure 10:**
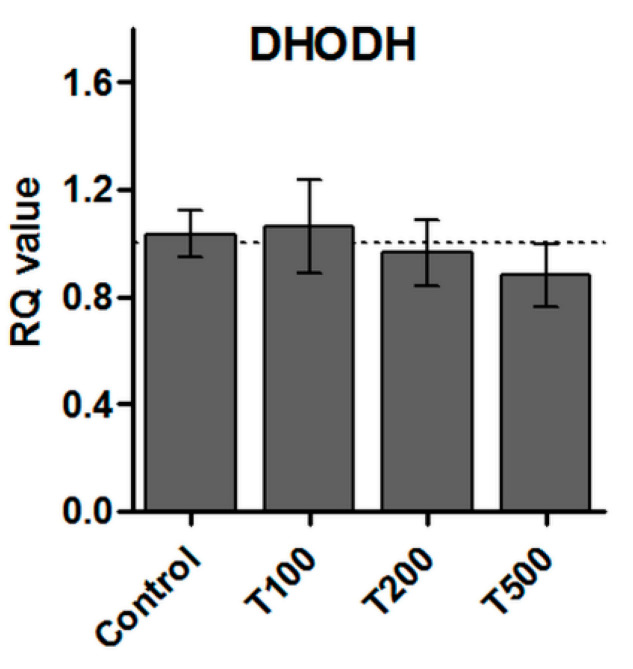
Relative mRNA expression level of *DHODH* in RPMI 8226 cells treated with teriflunomide (T) (100–500 µM) or DMSO as vehicle in control cultures for 24 h. *ACTB* and *18SN5* were used as reference genes. The obtained results were calculated as RQ values and presented as mean ± SD value of three independent experiments.

Relative gene expression assessment revealed that teriflunomide at all tested concentrations did not have any influence on DHODH gene expression. Teriflunomide did not change DHODH gene expression level as compared to the control (Figure 10).

### 2.8. Accumulation of Teriflunomide in RPMI 8226 Cells

Peaks of A771726 and warfarin (IS) were well resolved from each other and from other peaks in the matrix (Supplementary Materials Figures S1–S4). There were no endogenous substances in the each fraction that interfered with the analytical runs. The resulting retention times for teriflunomide (A771726) and the internal standard were averaged 5.7 and 8.8 min respectively. The results of HPLC analysis are shown in Table 1. Chromatographic analysis of individual cell fractions showed that the drug is present in the cytoplasm and nuclei of cells (in similar concentrations), but has not been found in mitochondria.

## 3. Discussion

Teriflunomide, active metabolite of leflunomide, inhibits the activity of DHODH, an enzyme crucial for de novo synthesis of pyrimidine nucleotides in activated lymphocytes [1,2]. As compared to other cells, hematopoietic cells require approximately an eight-fold higher levels of pyrimidine nucleotides for cell division. To completely meet these requirements, cells use both de novo as well as salvage pathways of pyrimidine nucleotide synthesis. Thus, teriflunomide inhibits activated lymphocytes in the G1 phase of the cell cycle and blocks their DNA synthesis [4]. Cancer cells also exhibit an increased demand for pyrimidine nucleotides, necessary for proper growth and proliferation [21,22]. Therefore, teriflunomide appears to be a promising anticancer drug.

One of the most advanced clinical trials using leflunomide as an anti-cancer drug concerns multiple myeloma. However, it has not yet been fully clarified what exact mechanism of action is responsible for the toxicity of this drug towards cancer cells and whether it is a mechanism dependent on DHODH inhibition. To demonstrate the importance of DHODH, cells were incubated simultaneously with teriflunomide and uridine. Uridine is produced by the biosynthesis of pyrimidine nucleotides [21]. Thus, the addition of uridine was expected to abolish the toxic effect of teriflunomide. Interestingly, simultaneous treatment with uridine (in both concentrations) did not revert the effect of teriflunomide on tested cells.

Similar studies on MM cell line were performed by Baumann et al. [23]. They noticed that teriflunomide induced apoptosis in cells and addition of uridine to cell culture medium reversed (only partially) observed apoptotic changes but merely at 200 µM of teriflunomide (48 h-treatment). These data clearly show that the effect depends on the concentration of the compound the addition of uridine to cell culture medium abolished cytotoxicity of teriflunomide in lower concentrations. The question remains if low concentrations of teriflunomide are of clinical importance. As one can see in the teriflunomide plasma pharmacokinetic summary of phase 1 clinical trial [19], Cmax_total_ ranges from 127.9 µM in patients receiving 20 mg leflunomide daily to as high as 695.2 µM in patients administrating 60 mg/day. Ultimately, Baumann et al. concluded that multiple myeloma cells are characterized by high uridine requirement and that the induction of apoptosis, as well as cell growth and cell cycle arrest induced by A771726, are not exclusively due to DHODH inhibition [23]. Similar observations have been noted for prostate cancer cells and chronic lymphocytic leukemia cells [17,24]. Interestingly, excess of uridine added to cell culture medium averted the teriflunomide effect in human T- and B-lymphocytes [25], normal human mast cells [26], and murine leukemia cells [27].

In the HepG2 liver cancer cell line, teriflunomide has an impact on DHODH transcription rate [7]. In our study, there were no significant changes in DHODH gene expression after teriflunomide treatment in the concentration range from 100 to 500 µM.

Cell cycle analysis results also indicate the DHODH- independent toxicity of teriflunomide in multiple myeloma cell line. At any tested concentrations of drug, we did not observe the inhibition of cell cycle in the G1 phase, which is typical for DHODH inhibition [5,17]. In contrast, after 100 µM teriflunomide treatment, cells were arrested in G2/M phase; however, in higher concentrations, a larger population of cells was observed in the subG1 phase corresponding to dead cells.

Annexin V-FITC/PI staining assay revealed cytotoxic effect of teriflunomide in RPMI 8226 cells. It may suggest that the cells observed in sub G1 phase of cell cycle were clearly apoptotic at higher drug concentrations. These results also suggest that teriflunomide at lower drug concentration—100 µM—inhibits proliferation of human multiple myeloma cells by promoting apoptosis and inducing the cell cycle arrest G2/M phase, which indicates possible DNA damage rather than its impaired synthesis [7].

Mitochondria play an essential role in programmed cell death (apoptosis) mainly by activating the intracellular apoptosis pathway (the so-called mitochondrial pathway). This is due to an increase in the mitochondrial membrane permeability caused by—e.g., overproduction of reactive oxygen species (ROS)—energy depletion or Ca^2+^ ions imbalance [28,29,30]. These processes are directly related to the formation of mitochondria permeability transition pore, allowing small molecules—e.g., cytochrome c and ions—to move freely along their concentration gradients, ultimately dissipating the mitochondrial membrane potential and disrupting the integrality of the mitochondrial outer membrane. Moreover, the production of ATP, glutathione, as well as NADH and NADPH are disrupted and transformed into oxidized forms and ROS are produced in excess during apoptosis [30,31,32].

Surprisingly, the results of mitochondrial membrane potential assay and level of cellular thiols indicate mitochondria-independent apoptosis due treatment with teriflunomide for 24 h in RPMI 8226 cells. No changes of mitochondrial membrane potential at any drug concentrations were observed. In addition, the level of cellular thiols also did not decrease after treatment. The cellular thiols, such as reduced glutathione (GSH) are involved in many processes in cells. They are key components involved in the maintenance of redox balance, which possess the ability to remove ROSs (free radicals), as well as playing a pivotal role in cell signaling, cell proliferation, and drug detoxification. Thus, the decreased level of cellular thiols is an early symptom of cell death progression in response to oxidative stress [30].

DHODH is associated with the respiratory complex and its loss leads to mitochondrial dysfunction. The oxidation of DHO to orotate is associated with the generation of superoxide from mitochondria [33], thus inhibition of DHODH is supposed to increase superoxide generation from mitochondria. However, there were no symptoms of oxidative stress in teriflunomide treated cells. Despite this fact, there have been several reports of oxidative stress reduction—e.g., in fetal human pulmonary arterial endothelial cells due teriflunomide treatment [34]—and its ability to preserve peripheral nerve mitochondria from oxidative stress [35].

As the obtained tests results did not show any involvement of mitochondria in the toxicity of teriflunomide, the localization of the compound in individual cell compartments was examined, using chromatographic methods. After 24 h of cell incubation, teriflunomide concentrations in the nuclear and cytoplasmic fractions were similar. Interestingly, no teriflunomide was found in the mitochondria. The results confirmed the mitochondria-independent mechanism of teriflunomide.

It has been shown in several studies that inhibition of protein tyrosine kinases activity in MM may be responsible for teriflunomide cytotoxic activity against cancer cells. Tyrosine kinases transduce signals from membrane receptors into the nucleus and stimulate multiple genes responsible for growth, differentiation, cell cycle control, adhesion, transmembrane and intracellular signaling, regulation of transcription and regulation of ion channels [36]. It has been noted that teriflunomide can inhibit PTK activity but mostly at higher concentrations—e.g., in mast cell, murine leukemia cell line (LSTRA), mouse embryonic fibroblasts cell line (Swiss 3T3) [6,11,26,37,38,39,40,41,42]. In the present study, the analysis of PTK activity revealed that only at highest drug concentration the inhibitory effect of teriflunomide was significant. Thus, the results confirm that teriflunomide exhibits the activity of tyrosine kinase inhibitor but in the highest clinically-achievable concentrations.

However, recently Buettner et al. [43] revealed that teriflunomide inhibits PIM family of serine/threonine kinases (PIMs) in MM cells, and in this way downregulates c-Myc expression and inhibits cell proliferation.

## 4. Materials and Methods

### 4.1. Cell Culture and Treatment

The study was conducted on multiple myeloma cell line- RPMI 8226 (catalog no. CCL-155, ATCC, Manassas, VA, USA) and skin fibroblast cell line- BJ (catalog no. CRL-2522, ATCC, USA). RPMI 8226 cells were grown on RPMI-1640 Medium (USA, ATCC) and BJ cells on EMEM Medium (USA, ATCC). The culture media were supplemented with 10% fetal bovine serum (Life Technologies, Carlsbad, CA, USA). Cells were cultured at 37 °C in 5% CO_2_-air. The media contained antibiotics such as penicillin and streptomycin (Sigma-Aldrich, St. Louis, MO, USA). In all assays, the cells were seeded into plates at concentration of 1.5 × 10^5^ cells/mL.

The cell cultures were treated with teriflunomide (A77 1726) (Selleckchem, München, Germany) in a wide range of concentrations (5–500 µM) or DMSO as vehicle in control cultures (maximal DMSO concentration < 0.5%). After the addition of compound, the cells were incubated for 24 h. The concentrations (100, 200, and 500 µM) of teriflunomide used in the further research had been selected after MTT assay and the assessment of cells morphology under the microscope.

### 4.2. Cytotoxicity Evaluation

Teriflunomide cytotoxicity, as well as the effect of uridine supplementation, were evaluated with the MTT assay using the MTT Cell Proliferation Assay Kit (Invitrogen, Waltham, MA, USA). The assay is based on the ability of living cells to reduce orange tetrazolium salt to water-insoluble purple formazan crystals. The cells were seeded into 96-well plates. MTT solution (4 mg/mL) was added to the cells after 24 h of incubation with tested compound. After 4 h of incubation, obtained formazan crystals were dissolved in DMSO. The solution absorbance was measured spectrophotometrically at wavelength of 540 nm, using PowerWave™ microplate spectrophotometer (Bio-Tek Instruments, Winooski, VT, USA). In the control, cells were treated with DMSO as a vehicle and blank presents results for a mixture of mediums without cells, MTT and DMSO. Each experiment was conducted three times and the measurements were performed in triplicates. IC_50_ value for teriflunomide was determined using the AAT Bioquest IC50 calculator.

To evaluate the morphology of cells, Nikon Eclipse TI optics inverted, a phase-contrast microscope was used. Microphotographs were prepared using NIS-Elements Imagine Software (Nikon Instruments Inc., Melville, NY, USA).

### 4.3. Analysis of Cell Cycle

The cell cycle phases at the single cell level were measured through the image analysis system NC-3000, using NC-3000 Two-step Cell Cycle Analysis Protocol (Denmark, Chemometec). The cells were seeded into 6-well plates. After 24 h of cells incubation with teriflunomide or DMSO as vehicle in control cultures, cells were harvested by centrifugation at 400× *g* at room temperature for 5 min. Next, cells were washed once with PBS, resuspended in 100 μL of lysis buffer (Solution 10) enriched with 10 μg/mL DAPI and incubated for 5 min at 37 °C. Then, 100 μL of stabilization buffer (Solution 11) was added to each sample. 10 μL of obtained cells suspension was applied into the NC-Slide and analyzed in NucleoCounter NC-3000. The experiment was conducted three times with measurements also being done in triplicate.

### 4.4. Apoptosis Detection

The assay was done using Annexin V/propidium iodide staining and NucleoCounter NC-3000. The cells were seeded into 6-well plates. After 24 h of incubation with teriflunomide or DMSO as vehicle in control cultures, cells were harvested by centrifugation at 400× *g* at room temperature for 5 min. Next, 100 μL of Annexin V binding buffer was added to the cells. Then, 2 μL of Annexin V-CF488A conjugate and 4 μL of Hoechst 33342 (10 μg/mL) were added to the cells and mixed carefully. The samples were incubated for 15 min at 37 °C, centrifuged at 400× *g* for 5 min, and were resuspended in 100 μL of Annexin V binding buffer supplemented with 10 μg/mL PI (prepared by add 2 μL Solution 16 to 100 μL binding buffer). 30 μL of obtained cells in suspension was loaded into the NC-slide and analyzed in NucleoCounter NC-3000. The experiment was conducted three times with measurements also being done in triplicate.

### 4.5. The Research of Oxidative Stress by the Level of Cellular Thiols

The level of cellular thiols in MM cells was measured using NC-3000 Vitality Assay (Denamark, Chemometec). The test is based on staining withVitaBright-48 that reacts with thiols and forms a fluorescent product—propidium iodide (PI)—which stains only dead cells, and acridine orange (AO) was used to stain all nucleated cells.

The cells were seeded into 6-well plates. After 24 h of cells incubation with teriflunomide or DMSO as vehicle in control cultures, cells were harvested by centrifugation at 400× *g* at room temperature for 5 min. Next, cells were mixed with solution 5 (containing all three dyes) at a 19/1 ratio. Then, 10 µL each of the cell mixture was loaded into the chambers of the NC-Slide and analyzed in NucleoCounter NC-3000. Each experiment was conducted three times with measurement in triplicate.

### 4.6. Analysis of the Mitochondrial Membrane Potential

The assessment of mitochondrial transmembrane potential (ΔΨm) was measured using JC-1 and DAPI fluorescent dyes (ChemoMetec, Denmark) with the fluorescence image cytometer NucleoCounter^®^ NC-3000™ system. The lipophilic cationic dye JC-1 accumulates in mitochondria in a potential-dependent manner. In healthy cells, the negative charge established by the intact mitochondrial membrane potential facilitates the accumulation of JC-1 in the mitochondrial matrix. At high concentrations, JC-1 forms red fluorescent aggregates. In turn, in dead cells, the mitochondrial potential collapses and JC-1 localizes in the cytosol in its monomeric green fluorescent form. The cells with collapsed mitochondrial potential exhibit a decrease in their red/green fluorescence intensity ratio. Cells were seeded into 6-well plates. After 24 h of cells incubation with teriflunomide or DMSO as vehicle in control cultures, cells were harvested by centrifugation at 400× *g* at room temperature for 5 min. Next, cells were suspended in 12.5 μL of JC-1 (final concentration: 2.5 μg/mL) and were incubated for 15 min at 37 °C. Then, the stained cells were centrifuged at 400× *g* for 5 min and washed twice with PBS. The obtained cell pellets were resuspended in 0.25 mL of DAPI and analyzed immediately using 8-chamber NC-Slides A8™ and the Mitochondrial Potential Assay protocol.

### 4.7. Activity of Protein Tyrosine Kinases

Measurement of protein tyrosine kinase (PTK) activity in MM cells was performed using a Universal Tyrosine Kinase Assay Kit (Takara Bio Inc., Kusatsu, Japan). The assay is based on the assessment of γ-phosphate residue transfer from ATP by PTK to peptide substrates which were immobilized on plate. All reagents, standards, and samples were prepared according to manufacturer instructions. The assay was conducted using a 96-well plate coated with PTK substrate, anti-phosphotyrosine (pY20)-horseradish peroxidase (HRP) antibody, and HRP substrate (TMBZ). Cell extract was added to the plate along with ATP solution to allow tyrosine phosphorylation. The sample solution was then removed and the plate thoroughly washed and blocked. Then pY20-HRP was added, incubated, and then replaced with TMBZ solution. The PTK activity of the samples was determined by comparing the absorbance of the sample at 450 nm with that obtained for the standard (via a plotted standard curve).

### 4.8. Quantitative Real-Time PCR Analysis (qRT-PCR)

Cells were seeded into 6-well plates. After 24 h of cells incubation with teriflunomide or DMSO as vehicle in control cultures, cells were harvested by centrifugation at 400× *g* at room temperature for 5 min. RNA was isolated according to the Chomczynski and Sacchi method [44]. Isolated RNA was reverse transcribed with an NG dART RT-PCR kit (EURx, Gdańsk, Poland) according to the manufacturer’s instructions. The qPCR was conducted using TaqMan^®^ Gene Expression Assays (USA, Life Technologies): DHODH- Hs00361406_mi, 18S-Hs99999901_s1, and *ACTB-*Hs03023943_g1, according to manufacturer’s instructions in a 7500 Fast Real-Time PCR System (ThermoFisher, USA). The reaction was carried out in triplicates. The relative expression of tested gene was determined by qRT-PCR and the ΔΔCt method using *ACTB and 18SRNA* as reference genes. The statistical analysis was performed with RQ values (relative quantification, RQ = 2^−∆ΔCt)^.

### 4.9. Statistical Analysis

The results were presented as mean ± SD and were analyzed with STATISTICA 13 software (StatSoft, Krakow, Poland). The values were compared using one-way analysis of variance (ANOVA) and post hoc multiple comparisons with Tukey’s honest significant difference test (Tukey’s HSD test). The results were considered statistically significant if the *p*-value was under 0.05.

### 4.10. HPLC Analysis

#### 4.10.1. Reagents and Chemicals

DMSO, acetonitrile, methanol, formic acid were purchased from POCH (Gliwice, Poland). Dulbecco’s phosphate-buffered saline (DPBS) was obtained from Mediatech, Inc. A Corning Subsidiary (Manassas, VA, USA). Warfarin and trifluoroacetic acid (TFA) were purchased from Sigma Aldrich (St. Louis, MO, USA). Milli-Q water (18.2 MΩ·cm) was prepared using Direct-Q 3 UV System, systems deliver both RO (Type 3) and ultrapure (Type 1) water directly from tap (Millipore, Bedford, MA, USA). All reagents and solvents have HPLC purity.

#### 4.10.2. Chromatographic Conditions

The apparatus used for the HPLC analysis consisted of Shimadzu model LC-20AD pump with a SPD-M20A, variable wavelength UV–visible detector, and a five-line-degasser (model DGU-20A5R). The column was heated using a Peltier-based column oven (model CTO-10ASvp). All apparatus elements were purchased from Shimadzu USA Manufacturing INC. (Canby, OR, USA). Separation of the analytes was performed on reverse-phased Phenomenex Kinetex C18 analytical column (5 µm spherical particles, pore size 100 Å, 250 × 4.6 mm) and C18 precolumn insert. The column temperature was maintained at 22 °C. The isocratic mobile phase consisted of acetonitrile, water and formic acid (50/49.8/0.2 *v*/*v*). A constant flow rate of 1.0 mL/min was used for the separation. UV detection was set at 293 nm.

#### 4.10.3. Standard Solution Preparation

Stock solution of teriflunomide (A771726) was prepared in DMSO, and then serially diluted with methanol to solutions of 0.25, 0.5, 1, 2.5, 5, 10, 25, 50, and 100 µg/mL. A working internal standard solution of warfarin (100 µg/mL) was prepared in methanol. Warfarin was added to each sample prior to chromatographic analysis. Stock solutions of standard and all samples were stored at −80 °C for up to one month.

#### 4.10.4. Sample Preparation

The cells were seeded into 25 cm^3^ flask. After 24 h of cell incubation with teriflunomide at 100 µM concentration, cells were harvested by centrifugation at 400× *g* at room temperature for 5 min. To prepare sample, 2 × 10^6^ cells were rinsed twice in cold DPBS. The pelleted cells were resuspended in 500 µL DPBS with 0.1% TFA and sonicated for 10 s. Warfarin was added to each sample as an internal standard (25 µL). The cell lysates were centrifuged at 800 *g* at 4 °C for 10 min, the obtained sediment was a nuclear fraction. The supernatant was transferred to another tube and centrifuged again at 15,000× *g* at 4 °C for 10 min, the obtained precipitate was a mitochondrial fraction. Hoestch 33342 (Thermo Fisher Scientific, Waltham, MA, USA) and MitoTracker^®^ Green FM (Thermo Fisher Scientific, Waltham, USA) were used to control fractionation quality. In the next step, residues were dissolved in 500 µL of a solution consisting of acetonitrile, water and formic acid (50/49.8/0.2 v/v). Prior to analysis, all the samples were filtered through a 0.45 µm vacuumed filter. A sample volume of 10 µL was injected onto the column. The concentration of each sample was determined by means of a linear equation calculated from the surface area.

The HPLC system was calibrated with intact teriflunomide. The assay was validated by determining linearity with solutions of teriflunomide in the appropriate concentration range. The linearity ranged from 100 ng to 100 µg and the measure of error of analysis was 1%.

## 5. Conclusions

The aforementioned results indicate DHODH- and mitochondria-independent mechanism of leflunomide toxicity against RPMI 8226 multiple myeloma cell line. Leflunomide, as a potential anti-cancer drug, is considered primarily as an inhibitor of DHODH. Nevertheless, the recognition of the mechanism of its action independent of the synthesis of pyrimidines, which makes it an effective anti-myeloma drug, may allow for the development of new therapeutic strategies in the treatment of myeloma and other cancers.

## Figures and Tables

**Table 1:** Concentration of teriflunomide (A771726) in each cell fraction and culture medium.

	Concentration (µg/mL)			
	RPMI-1640 Culture Medium	Cytoplasm Fraction	Nuclear Fraction	Mitochondrial Fraction
**Teriflunomide**	17.157	2.104	1.975	peak not detected

## Data Availability

The data presented in this study are available on request from the corresponding author.

## Supplementary Materials

The following are available online, Figure S1. A Chromatogram of culture media RPMI-1640 (control without A771726), B chromatogram of culture media RPMI-1640 from cells culture incubated with A771726. Figure S2. A Chromatogram of cell cytoplasm (control without A771726), B chromatogram of cell cytoplasm from cells culture incubated with A771726. Figure S3. A Chromatogram of nuclear fraction (control without A771726), B chromatogram of nuclear fraction from cells culture incubated with A771726. Figure S4. A Chromatogram of mitochondrial fraction (control A771726), B chromatogram of mitochondrial fraction from cells culture incubated with A771726.

## Sample Availability

Samples of the compounds are not available from the authors.
