# Supplementary material for: The Mitochondria-Independent Cytotoxic Effect of Leflunomide on RPMI-8226 Multiple Myeloma Cell Line
Source: Molecules. 2021 Sep 17;26(18):5653. doi: 10.3390/molecules26185653 (PMC8469018; doi:10.3390/molecules26185653)
Supplement: Supplementary file 1 [file molecules-26-05653-s001.zip › molecules-1358871-supplementary.pdf]

THE MITOCHONDRIA-INDEPENDENT CYTOTOXIC EFFECT OF LEFLUNOMIDE ON RPMI-8226 MULTIPLE MYELOMA CELL LINE

Grzegorz Adamczuk[1],* Ewelina Humeniuk[1], Magdalena Iwan[2], Dorota Natorska-Chomicka[2], Kamila Adamczuk[3],and Agnieszka Korga-Plewko[1]

[1] Independent Medical Biology Unit, Faculty of Pharmacy, Medical University of Lublin,PL-20093 Lublin, Poland; grzegorzadamczuk@umlub.pl (G.A); ewelinahumeniuk@umlub.pl (E.H); agnieszkakorga@umlub.pl (A.K.-P).

[2] Chair and Department of Toxicology, Faculty of Pharmacy, Medical University of Lublin, PL-20093 Lublin, Poland; magda.iwan@umlub.pl (M.I); dorota.chomicka@umlub.pl (D.N-CH).

[3] Chair and Department of Biochemistry and Molecular Biology, Faculty of Medicine, Medical University of Lublin, PL-20093 Lublin, Poland, kamilaadamczuk@umlub.pl (K.A)

* Correspondence: grzegorzadamczuk@umlub.pl; Tel: 48-81-448-65-20

Figures

Figure S1. A Chromatogram of culture media RPMI-1640 (control without A771726), **B** chromatogram of culture media RPMI-1640 from cells culture incubated with A771726.

Figure S2. A Chromatogram of cell cytoplasm (control without A771726), **B** chromatogram of cell cytoplasm from cells culture incubated with A771726.

Figure S3. **A** Chromatogram of nuclear fraction (control without A771726), **B** chromatogram of nuclear fraction from cells culture incubated with A771726.

Figure S4. **A** Chromatogram of mitochondrial fraction (control A771726), **B** chromatogram of mitochondrial fraction from cells culture incubated with A771726.

A)

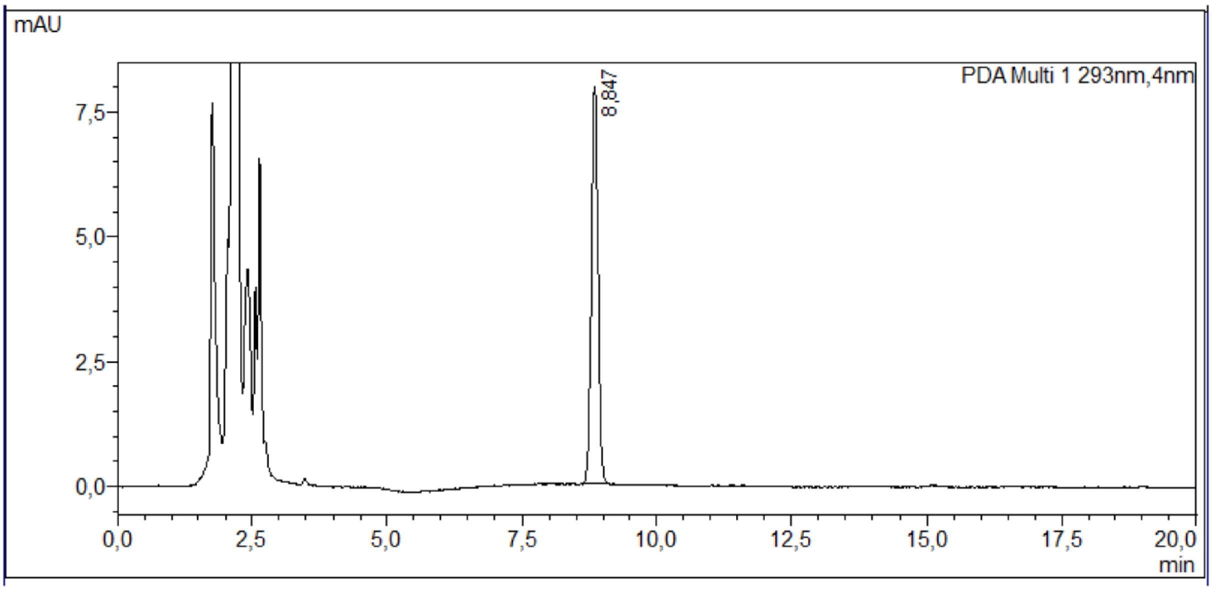

B)

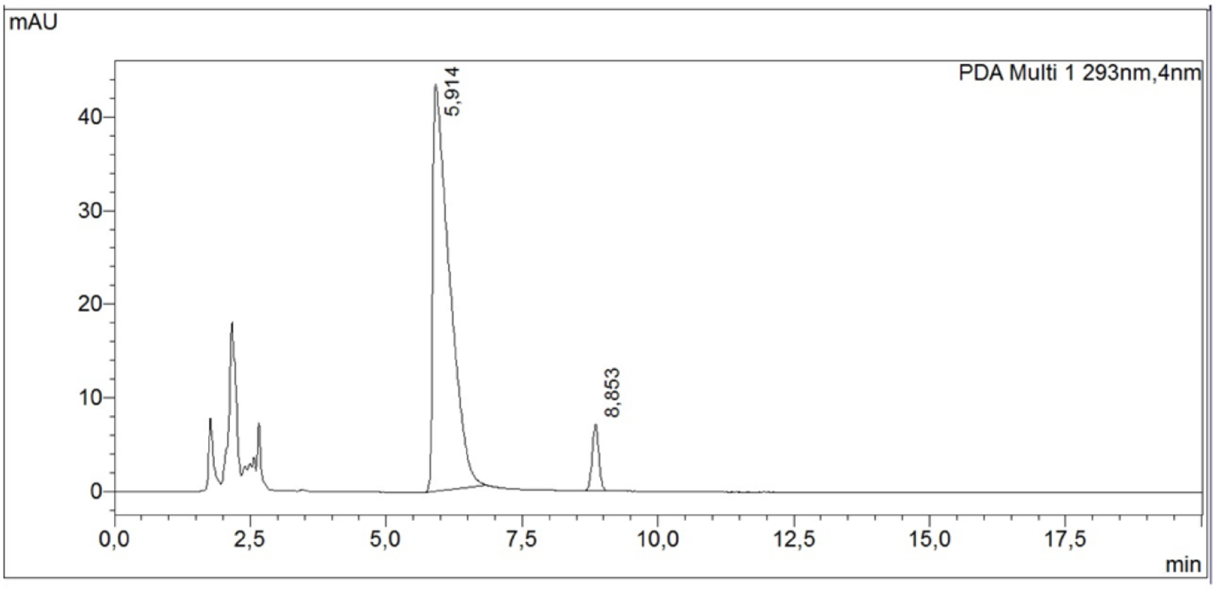

Figure S1. A Chromatogram of culture media RPMI-1640 (control without A771726), **B** chromatogram of culture media RPMI-1640 from cells culture incubated with A771726.

A)

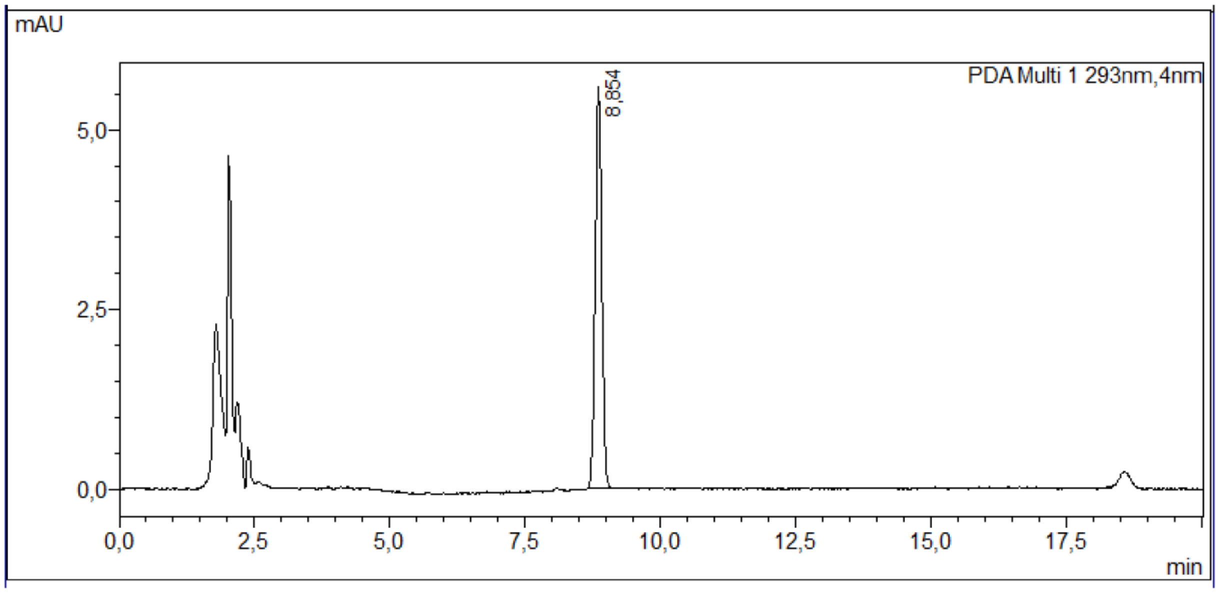

B)

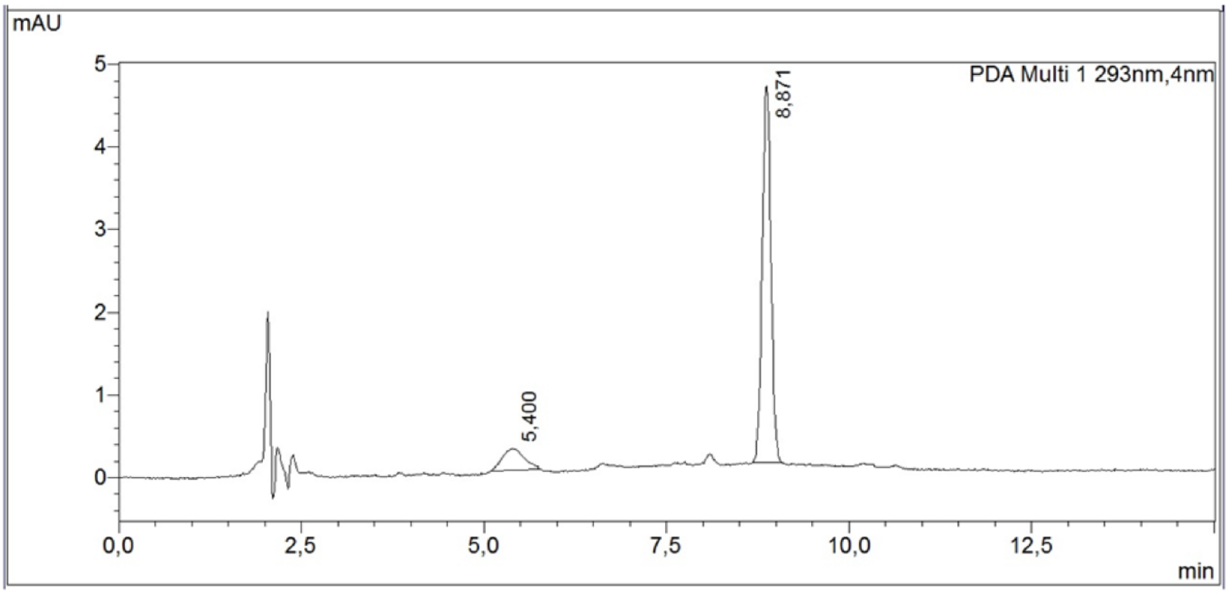

Figure S2. A Chromatogram of cell cytoplasm (control without A771726), **B** chromatogram of cell cytoplasm from cells culture incubated with A771726.

A)

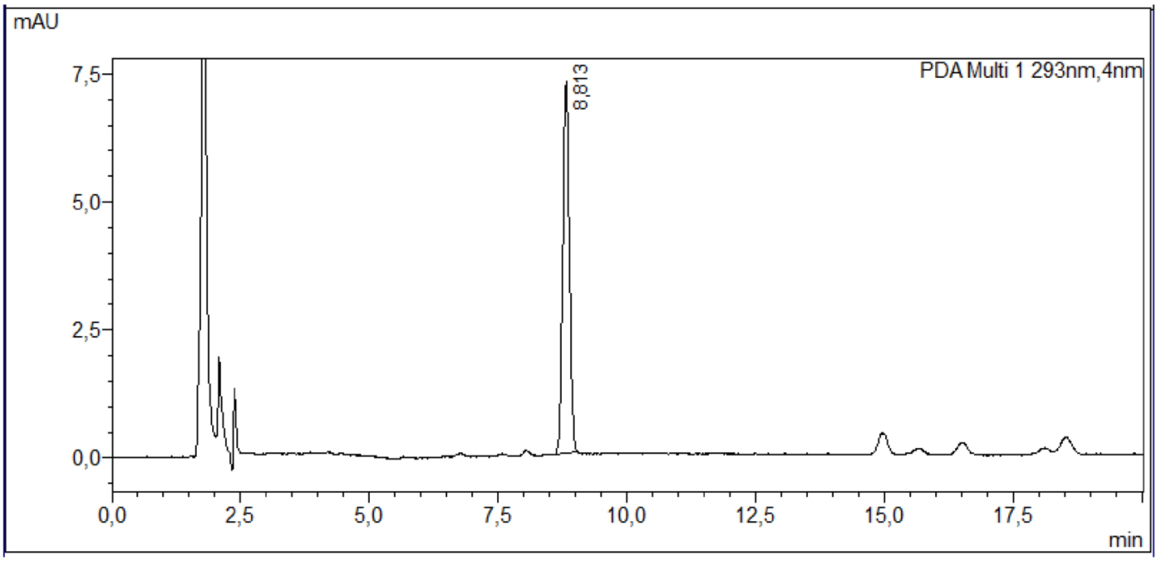

B)

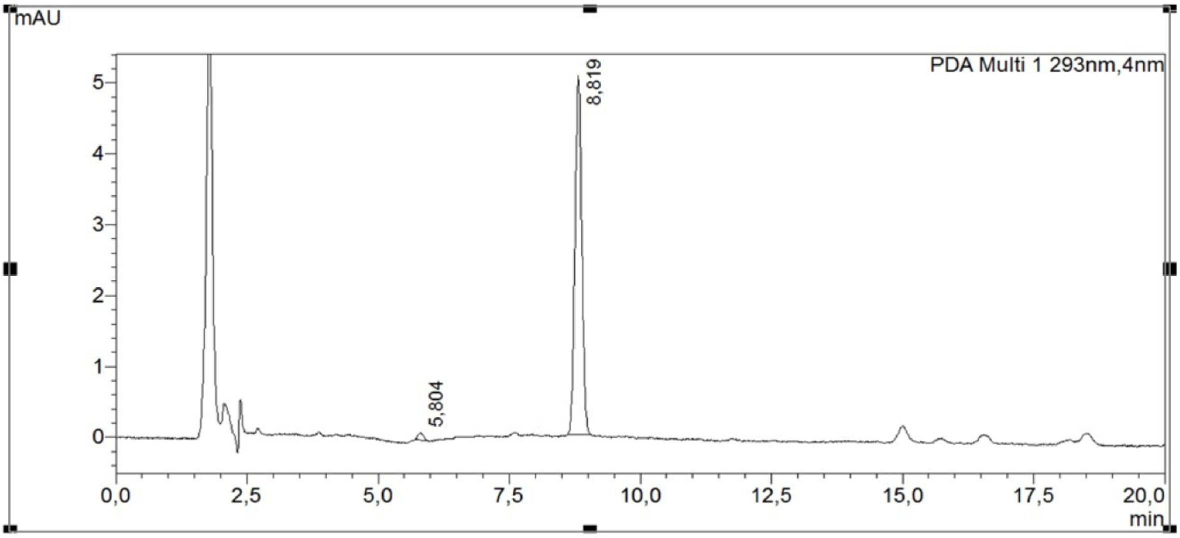

Figure S3. **A** Chromatogram of nuclear fraction (control without A771726), **B** chromatogram of nuclear fraction from cells culture incubated with A771726.

A)

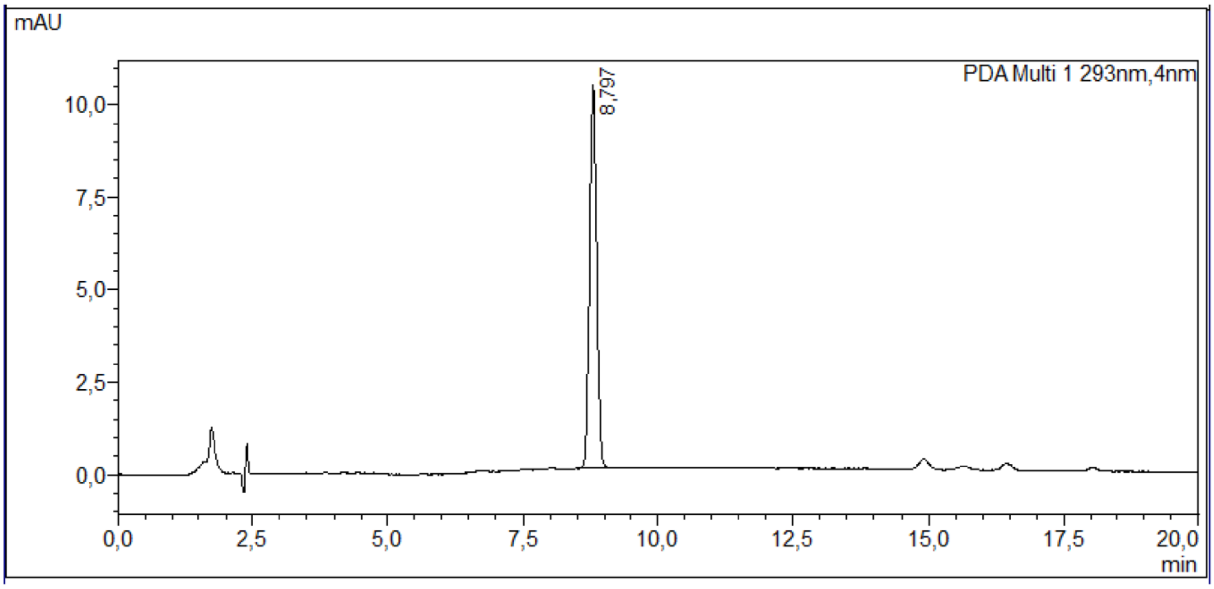

B)

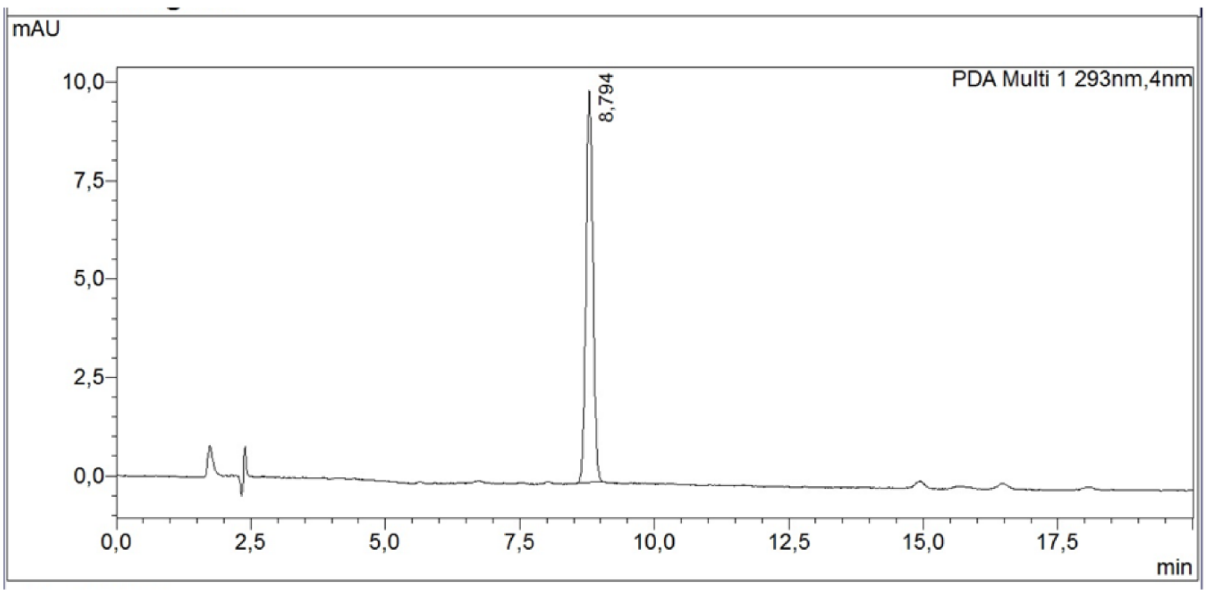

Figure S4. **A** Chromatogram of mitochondrial fraction (control A771726), **B** chromatogram of mitochondrial fraction from cells culture incubated with A771726.
